# Association of plasma neurofilament light protein concentration with sleep disturbance after intracerebral hemorrhage

**DOI:** 10.3389/fneur.2025.1482808

**Published:** 2025-04-30

**Authors:** Peng Xu, Jinlei Yang, Xin Zhao, Fang Liu, Qiang Liu, Handong Wang

**Affiliations:** ^1^Department of Neurosurgery, The Affiliated BenQ Hospital of Nanjing Medical University, Nanjing, China; ^2^Department of Neurosurgery, Affiliated Jinling Hospital, School of Medicine, Nanjing University, Nanjing, Jiangsu, China; ^3^Department of Cardiothoracic Surgery, Jinling Hospital, Medical School of Nanjing University, Nanjing, China

**Keywords:** biomarker, plasma NfL, intracerebral hemorrhage, sleep disorder, prognosis

## Abstract

**Background:**

Intracerebral hemorrhage (ICH) represents a critical subtype of stroke characterized by substantial morbidity and mortality. Emerging research indicates that neurofilament light protein (NfL), a biomarker indicative of neuronal damage, may offer valuable prognostic information regarding outcomes and recovery trajectories post-ICH. This study seeks to elucidate the relationship between plasma NfL (pNfL) concentrations and long-term patient outcomes, with a particular focus on sleep disturbances following ICH.

**Methods:**

We conducted a cohort study comprising 26 healthy controls and 49 patients who had experienced ICH. The Glasgow Coma Scale (GCS) was assessed upon admission. Plasma samples were collected at admission and on 3, 7, and 14 days post-ICH. Then pNfL levels were quantified using Enzyme-Linked Immunosorbent Assay (ELISA). Clinical outcomes were evaluated at 6 months post-ICH using the Glasgow Outcome Scale-Extended (GOSE) and the Pittsburgh Sleep Quality Index (PSQI). Receiver operating characteristic (ROC) curves and the areas under the ROC curves (AUC) were utilized to determine the accuracy of hemorrhage volume and pNfL levels in identifying sleep disturbances.

**Results:**

pNfL levels were elevated in patients with ICH compared to healthy controls. Longitudinal analysis indicated an increasing trend in pNfL levels over the initial 7 days post-admission. pNfL levels demonstrated an AUC for distinguishing ICH patients from controls (admission for 0.92, post-ICH 3d for 0.98). In ICH patients, pNfL levels showed a positive correlation with hemorrhage volume and PSQI, and a negative correlation with GCS and GOSE. The AUCs for pNfL levels and hemorrhage volume, which were indicative of sleep disturbances, were 0.82 and 0.75, respectively. Furthermore, the combined assessment of pNfL levels and hemorrhage volume exhibited superior predictive performance compared to the evaluation of each factor individually.

**Conclusion:**

pNfL represents a promising biomarker for predicting functional outcomes and evaluating sleep disturbances in patients following ICH. Elevated levels of NfL at admission are associated with poorer prognoses and increased sleep-related issues, indicating that monitoring pNfL could be valuable for prognostication and the implementation of targeted interventions.

## Introduction

Intracerebral hemorrhage (ICH), the most prevalent form of hemorrhagic stroke, constitutes approximately 10–15% of all stroke cases but is associated with higher morbidity and mortality rates compared to ischemic strokes (1). ICH is characterized by the rupture of a blood vessel, resulting in hemorrhage within the brain parenchyma and subsequent neuronal tissue damage. This causes Hemiplegia (2), aphasia (3), sleep disturbances (4), Cognitive impairment (5), even death. Notably, nearly half of ICH patients experience sleep disorders, which significantly impede their recovery and diminish their quality of life (6). Sleep disturbances have the potential to exacerbate neurological outcomes by contributing to increased blood pressure variability, impaired neural repair processes, and diminished overall resilience to subsequent brain injuries (7). Despite the high prevalence and significant impact of sleep disturbances on post-stroke recovery, these issues are frequently underdiagnosed and undertreated. Early identification and management of sleep disturbances are therefore crucial (8). Emerging research on biomarkers of neural damage, which has garnered increasing attention, may prove instrumental in the early detection of sleep disorders.

Due to limited tissue specificity and susceptibility to external influences, certain biomarkers, such as S100B and neuron-specific enolase, may not be optimal (9). Consequently, the identification of reliable biomarkers is essential for predicting clinical outcomes and sleep disorders in the late phase following ICH.

Neurofilament light chain (NfL) is a cytoskeletal protein predominantly localized in large-caliber myelinated axons. NfL is released into the cerebrospinal fluid and peripheral blood following neuronal injury such as that caused by stroke (10), traumatic brain injury (11), amyotrophic lateral sclerosis (12), and other central nervous system diseases, thereby, it as a promising biomarker for neuronal damage (13, 14). Elevated levels of NfL have been correlated with a range of neurological disorders, including multiple sclerosis, Alzheimer’s disease, stroke, and traumatic brain injury, therefore suggesting its potential utility in assessing the severity of neural injury (15). Previous studies have demonstrated a high prevalence of sleep disorders in up to 78% of stroke patients (16), which not only exacerbate the risk of post-stroke anxiety and depression but also exhibit a correlation with poor neurological functional outcomes after stroke (17, 18). Nevertheless, the role of NfL as a biomarker for sleep disturbances and clinical outcomes following ICH remains inadequately understood.

In this prospective observational study, the objective was to evaluate pNfL concentrations in patients following ICH, alongside assessments using the Glasgow Outcome Scale-Extended (GOSE) and the Pittsburgh Sleep Quality Index (PSQI), and to investigate potential correlations among these variables.

## Materials and methods

### Participants

The Ethics Committee of the Affiliated BenQ Hospital of Nanjing Medical University, Nanjing, China, granted approval for the inclusion of human subjects in this study (Grant Number:2024-KL024) All methodologies were executed in strict adherence to the pertinent guidelines and regulations established by the Ethics Committee. Patients admitted to the neurosurgery department of the Affiliated BenQ Hospital of Nanjing Medical University, Nanjing, China, between January 2024 and May 2024 were screened for eligibility. Inclusion criteria encompassed patients (age:18 to 60 years), within 24 h of ICH symptom onset, with image of ICH from non-contrast head computed tomography (CT) scan in the emergency room, with ICH volume quantified using the ABC/2 method. The exclusion criteria included a history of clinically symptomatic intracerebral hemorrhage (ICH), prior traumatic head injuries with residual deficits, pre-existing central or peripheral nervous system diseases, oncological conditions, and pregnancy. Upon admission to the emergency room, all patients underwent a standard non-contrast head CT scan as part of routine care to exclude ischemic stroke. The control group comprised individuals aged 18 to 60 years who were confirmed to be free of nervous system diseases and other severe conditions based on their examination outcomes.

### Blood sampling and plasma neurofilament light

Blood samples were obtained via venepuncture of the peripheral veins and collected into tubes containing the anticoagulant ethylenediaminetetraacetic acid (EDTA) at admission, and on days 3, day 7 and day 14 post-ICH between January 2024 and May 2024. Subsequently, the samples were centrifuged at 3000 g for 10 min at 4°C within 1 h of collection to isolate the plasma. The plasma was then transferred into EP tubes and immediately stored at −80°C until further analysis.

The concentration of plasma neurofilament light chain (NfL) was measured using a human NfL ELISA kit (biotechnology corporation, Jiangsu, CN), which has an analytical sensitivity of 8 pg./mL for NfL. In brief, 50 μL of standards and diluted samples (5-fold dilution) were added to a pre-coated microplate and incubated at 37°C for 60 min. Subsequently, 50 μL of NfL detector antibody was introduced into each well and incubated at 37°C for an additional 30 min. Following this, 100 μL of TMB substrate was added and incubated for 10 min at 37°C in the dark. This was followed by the addition of 50 μL of stop solution. The absorbance in each well was then measured using a Synergy HTX plate reader at an optical density of 450 nm. Background noise was corrected by subtracting the average absorbance value of the zero standard (sample diluent only). The concentration of NfL was subsequently calculated from the standard curve.

### Outcomes

The Glasgow Outcome Scale-Extended (GOSE) and the Pittsburgh Sleep Quality Index (PSQI) were evaluated through interviews or telephone assessments at 6 months post- ICH. Data collection for the GOSE and PSQI interviews was standardized according to a manual specifically designed for ICH to assess functional recovery. The GOSE scores were determined as previously described in the literature. Both the GOSE and PSQI questionnaires were centrally scored through a rating process. The GOSE scores were categorized as follows: dead (GOSE = 1), vegetative state (VS, GOSE = 2), lower severe disability (SD−, GOSE = 3), upper severe disability (SD+, GOSE = 4), and lower moderate disability (MD−, GOSE = 5), upper moderate disability (MD+, GOSE = 6), lower good recovery (GR−, GOSE = 7), and upper good recovery (GR+, GOSE = 8). Furthermore, a PSQI score of ≤ 10 signifies high sleep quality, whereas a score of > 10 denotes low sleep quality.

### Statistics

Statistical analyses were conducted utilizing SPSS version 26 and MedCalc version 22. Figures were generated using GraphPad Prism version 8. The normality of data distribution was evaluated using the Shapiro–Wilk test. Continuous variables are reported as mean ± standard deviation or median with interquartile range, while categorical variables are presented as frequencies and percentages. Unpaired Student’s t-tests were performed to assess differences in pNfL levels between control subjects and patients at various time points (at admission and day 3, day 7, and day 14 post- ICH). The tendency of changes in pNfL levels over the first 7 days post-ICH was examined using paired t-tests. The area under the curve (AUC) was employed to evaluate the discriminatory capacity for ICH, with the optimal cut-off point determined as the pNfL value yielding the highest correct classification rate. Additionally, the relationship between pNfL concentrations and hemorrhage volume was investigated using Pearson correlation analysis and linear regression. The associations between pNfL concentrations, GCS, GOSE, and PSQI were evaluated using Spearman’s rank correlation test and binary logistic regression models.

## Results

### Demographic and clinical characteristics

The clinical and demographic characteristics of the ICH patients are summarized in Table 1. There were no significant differences in age (47.9 ± 8.8 years versus 44.3 ± 10.0 years; *p* = 0.44) between the ICH patients and the control group. Male patients constituted 85.7% of the patient cohort. Upon admission, GCS scores were recorded, indicating mild disorder of consciousness in 13 patients (26.5%), moderate disorder of consciousness in 15 patients (30.6%) and severe disorder of consciousness in 21 (42.9%). Bleeding locations included the basal ganglia (8%), thalamus (29%), and lobar regions (26%). The mean hematoma volume was 35.2 ± 13.1 mL. Computed tomography (CT) scans revealed that 8 patients exhibited hemorrhage extending into the ventricles. A total of 46 patients (93.9%) underwent surgical hematoma evacuation. Additionally, 69.4% of the patients had a prior history of hypertension. At the 6-month follow-up, 2 patients (4.1%) had died (both within 30 days of injury), 17 patients had an unfavorable outcome [including vegetative state (VS, 2%), severe disability (SD−, 14.3%), and moderate disability (SD+, 18.4%)], and 30 patients had a favorable outcome [including moderate disability (MD−, 12.2%), mild disability (MD+, 12.2%), good recovery (GR−, 14.3%), and good recovery (GR+, 22.4%)], as assessed by the Glasgow Outcome Scale (GOS) (Table 1). Regarding the Pittsburgh Sleep Quality Index (PSQI), 18 patients (39.1%) exhibited the highest sleep quality, with PSQI scores ranging from 0 to 5. Conversely, 2 patients (4.4%) were classified in the poorest sleep quality category, with PSQI scores between 16 and 21. The distribution of PSQI scores among the remaining patients was as follows: 14 patients scored between 6 and 10, and 12 patients scored between 11 and 15.

**Table 1:** Demographic and clinical characteristics of the patients with ICH.

Variable	Result
Age, years	47.9 ± 8.8
Female, *n* (%)	7 (14.3%)
Glasgow Coma Scale, *n* (%)	
Mild (13–15)	13 (26.5%)
Moderate (9–12)	15 (30.6%)
Severe (3–8)	21 (42.9%)
Hemorrhage location, *n* (%)	
Basal ganglia	34 (69.4%)
Thalamus	11 (22.4%)
Lobar	4 (8.2%)
Hematoma volume, mL	35.2 ± 13.1
Ventricular extension, *n* (%)	8 (16.3%)
Surgical hematoma evacuation, *n* (%)	46 (93.9%)
Hypertension, *n* (%)	34 (69.4%)
The score of GOSE	5.4 ± 2.1
Dead	2 (4.1%)
VS	1 (2%)
SD−	7 (14.3%)
SD+	9 (18.4%)
MD−	6 (12.2%)
MD+	6 (12.2%)
GR−	7 (14.3%)
GR+	11 (22.4%)
The score of PSQI, *n* (%)	
0–5	18 (39.1%)
6–10	14 (30.4%)
11–15	12 (26.1%)
16–21	2 (4.4%)

Data presented as number (%), GOSE, Glasgow Outcome Scale-Extended; PSQI, Pittsburgh sleep quality index; VS, vegetative state; SD−, lower severe disability; SD+ upper severe disability; MD−, lower moderate disability; MD+, upper moderate disability; GR−, lower good recovery; GR+, upper good recovery.

### pNfL was elevated following ICH in patients

**Figure 1:**
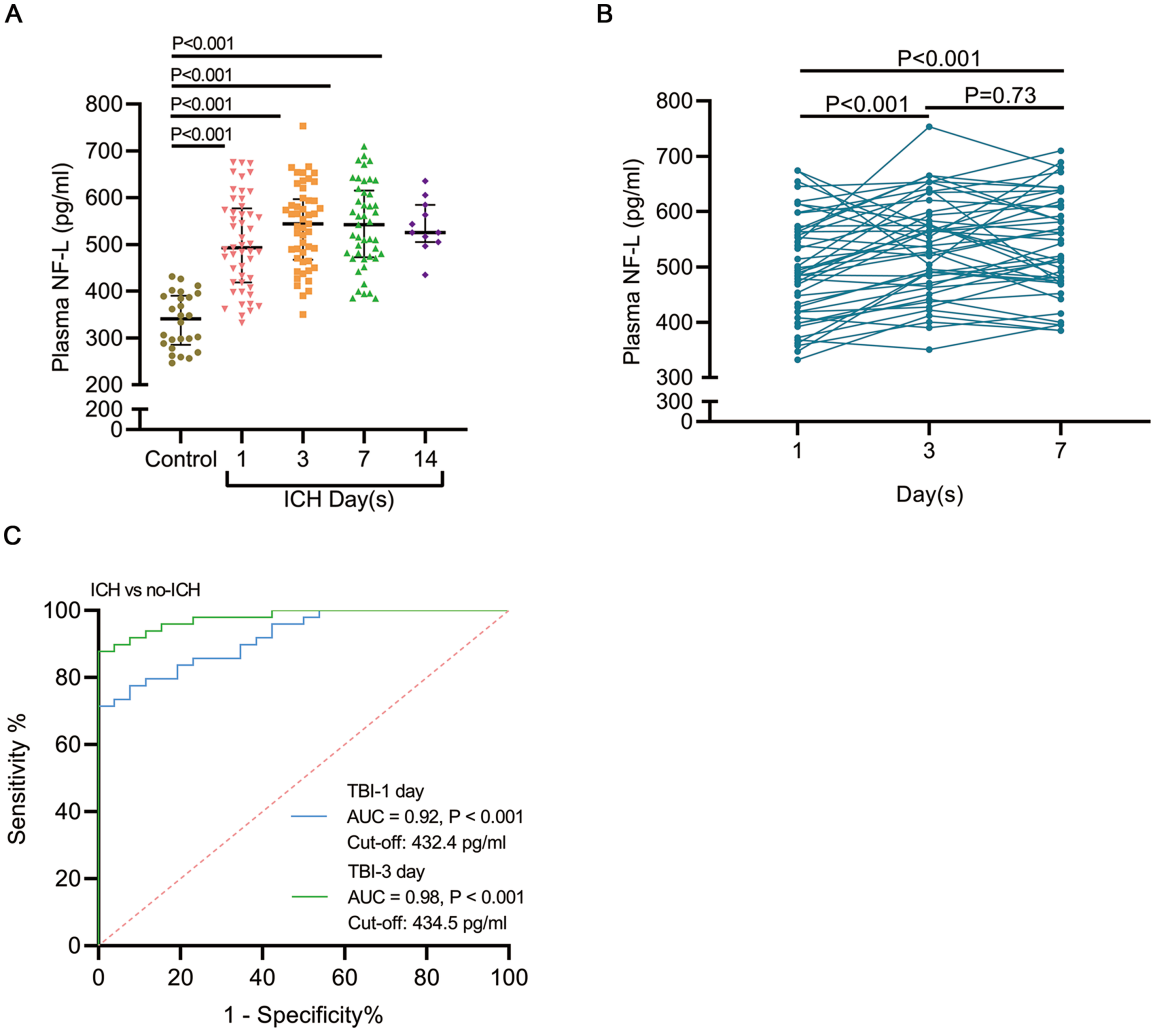
pNfL levels are elevated in patients following ICH. **(A)** A comparison of pNfL levels in ICH patients within 1 day of ictus with those in healthy individuals is presented. Individual patient data are depicted, with lines indicating the median and interquartile range. **(B)** Blood samples were collected from 45 patients at admission, and on days 3 and 7 post-ICH; the continuous changes in pNfL levels at these time points were evaluated. **(C)** The area under the receiver operating characteristic curve (AUC) for pNfL levels at admission and on day 3 post-ICH was compared to the control group. Data are presented as the median ± interquartile range. The statistical significance of the differences was assessed using the unpaired t-test (comparing healthy individuals and patients) and the paired t-test (comparing values at admission, day 3, and day 7). The sample sizes were *n* = 26 for controls and *n* = 49 for ICH patients. The AUC and *p* values are illustrated in the panel.

In comparison to healthy controls, who had a mean plasma neurofilament light chain (pNfL) level of 336.6 ± 58.9 pg./mL, patients with intracerebral hemorrhage (ICH) demonstrated significantly elevated pNfL levels, averaging 503.5 ± 98.6 pg./mL (*p* < 0.001; Figure 1A). To further elucidate the temporal changes in pNfL levels from the acute to subacute phases, we conducted serial measurements of pNfL at admission, on day 3, and on day 7 in a cohort of 45 patients. Interestingly, pNfL levels at day 3 post-ICH (537.0 ± 88.6) were significantly higher than those measured at admission (497.5 ± 94.0). However, the difference between pNfL concentrations at day 3 post-ICH and day 7 post-ICH (540.3 ± 91.0) was not statistically significant (Figure 1B). pNfL effectively distinguished ICH patients from controls. As a proof-of-concept, we evaluated the diagnostic utility of pNfL for ICH by analyzing the area under the curve (AUC) for pNfL levels at different time points post-ICH compared to the control group. The AUC for pNfL at day 3 post-ICH increased to 0.98, compared to 0.92 at admission (Figure 1C). The optimal individual cut-off levels for plasma neurofilament light (pNfL) were determined to be 432.4 pg./mL (Youden index, 0.92) and 434.5 pg./mL (Youden index, 0.98) at the time of admission. In comparison to the admission data, the data collected on day 3 demonstrated greater significance for clinical diagnosis. Furthermore, the application of pNfL cut-off levels at admission resulted in a sensitivity of 71.4% and a specificity of 100%.

### pNfL correlated with the disease severity of ICH in the acute phase

**Figure 2:**
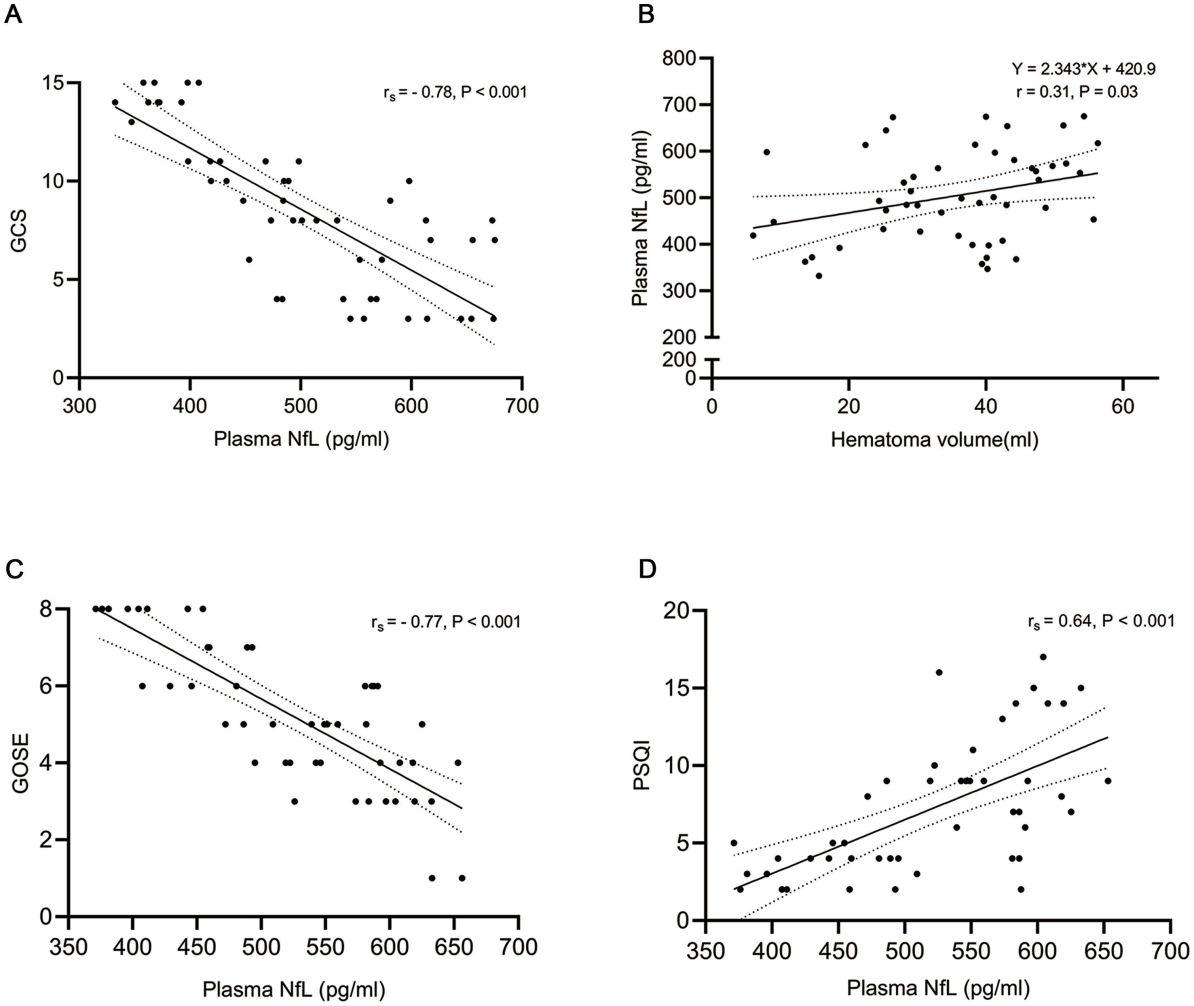
The correlation of plasma neurofilament light (pNfL) with the Glasgow Coma Scale (GCS), hematoma volume, Glasgow Outcome Scale Extended (GOSE), and Pittsburgh Sleep Quality Index (PSQI) was analyzed. Specifically, **(A)** shows the correlation between pNfL levels on day 1 and GCS (*n* = 49), **(B)** depicts the correlation between pNfL levels on day 1 and hematoma volume (*n* = 49), and **(C)** illustrates the correlation between mean pNfL levels within 14 days and GOSE. **(D)** The correlation between mean pNfL levels within 14 days and the PSQI was assessed. Associations between pNfL and GCS, GOSE, and PSQI were evaluated using Spearman’s test, while associations between pNfL and hematoma volume were assessed using Pearson’s test. The r and p values are presented in the figure.

To elucidate the relationship between pNfL levels and the severity of acute brain tissue damage following ICH, we collected blood samples, conducted computed tomography (CT) scans at admission, and assessed the patients’ level of consciousness using the Glasgow Coma Scale (GCS). Firstly, pNfL exhibited a negative correlation with the GCS score, as determined by Spearman’s correlation (r_s_ = −0.78, *p* < 0.001; Figure 2A). Linear correlation analysis revealed a significant association between pNfL levels and hematoma volume, measured using the ABC/2 formula upon admission following ICH (r = 0.31, *p* = 0.03; Figure 2B). Furthermore, multivariate linear regression analysis indicated that pNfL levels were positively correlated with hematoma volume both at admission and on day 7 post-ICH (*p* = 0.004 and *p* = 0.02, respectively; Table 2). However, no such association was observed when pNfL levels were measured on day 3 post-ICH, either in unadjusted analyses or in multivariable models adjusted for age, sex, hypertension, hemorrhage location, ventricular extension, and surgical hematoma, using linear regression models. Subsequently, we investigated whether hemorrhage location, ventricular extension, and surgical treatment influenced pNfL levels. In our stratified analyses, multivariate linear regression revealed that the association between pNfL and hemorrhage location (basal ganglia, thalamus, and lobar) was significant only after adjustment (*p* = 0.008; Table 2).

**Table 2:** Association of NfL concentrations with hematoma volume.

		Univariable analysis		Multivariable analysis	
Variable types	*N*	β (95% CI)	*p*-value	β (95% CI)	*p*-value
Time (pNfL)					
Admission	49	0.31 (0.24–4.45)	0.03	0.56 (1.42–7.13)	0.004
3 day post-ICH	49	0.17 (−0.76–3.05)	0.23	0.30 (−0.26–4.20)	0.08
7 day post-ICH	45	0.28 (−0.13–3.87)	0.06	0.49 (0.51–6.13)	0.02
Hemorrhage location	49	−0.21 (−75.90–12.68)	0.158	−0.40 (−87.56–14.35)	0.008
Ventricular extension	49	0.06 (−62.25–92.50)	0.69	−0.12 (−95.80–45.83)	0.48
Surgical hematoma	49	0.003 (−93.51–95.75)	0.98	−0.32 (−178.18–9.27)	0.07

### pNfL correlated with long-term outcomes of patients with intracerebral hemorrhage

**Figure 3:**
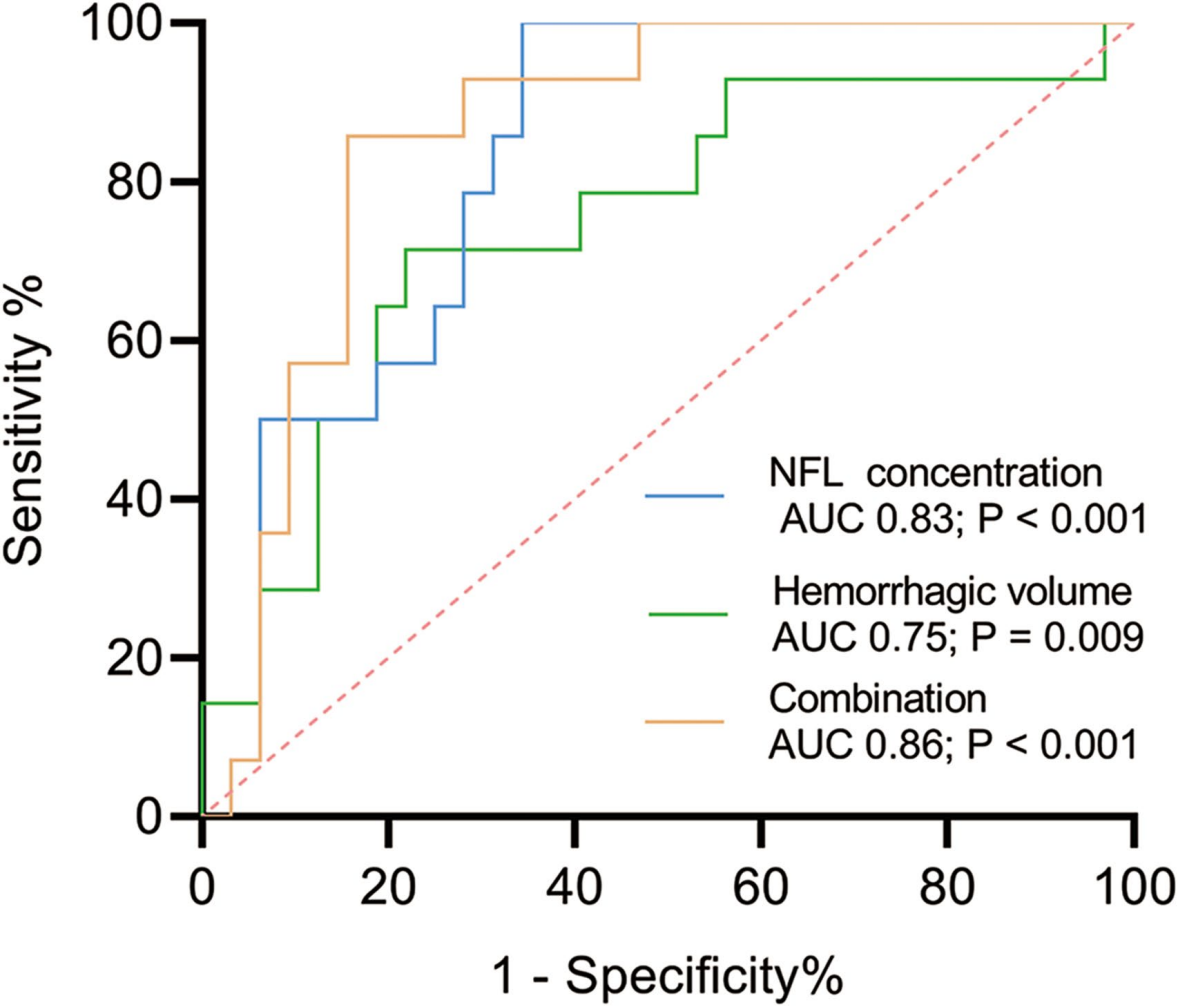
ROC curves were generated for pNfL and hematoma volume to detect ICH. The AUC values for pNfL and hemorrhage volume, indicating the occurrence of sleep disturbance, were 0.82 and 0.75, respectively. The AUC for the combination of pNfL and hemorrhage volume was 0.86, demonstrating superior predictive performance compared to hematoma volume alone. AUC and *p* values are shown in the panel.

The Glasgow Outcome Scale Extended (GOSE) and the Pittsburgh Sleep Quality Index (PSQI) were assessed at 6 months post-ICH, facilitating the examination of the relationship between pNfL levels and long-term ICH outcomes. Mean pNfL levels measured within 14 days post-ICH were significantly correlated with GOSE and PSQI scores, as determined by Spearman’s correlation (r_s_ = − 0.77, *p* < 0.001; r_s_ = 0.64, *p* < 0.001, respectively; Figures 2C,D). Binary logistic regression models were employed to investigate the association between pNfL levels and sleep disorders. The analysis revealed a positive correlation between pNfL levels and PSQI scores following ICH (Univariable analysis, *p* = 0.003; Multivariable analysis, *p* = 0.007; Table 3), indicating a relationship with poorer clinical outcomes. In subgroup analyses, ventricular extension was positively correlated with the PSQI score of patients (*p* = 0.02; Table 3) after adjusting for potential confounding factors such as age, sex, and hypertension. To further evaluate the predictive ability of NfL levels within 14 days for a PSQI score greater than 10 at 6 months post-ICH, we estimated the area under the ROC curve (AUC) with and without pNfL in the binary logistic regression model. The inclusion of NfL in the model for single hemorrhage volume increased the AUC from 0.75 to 0.86 at 6 months (*p* = 0.096; Figure 3).

**Table 3:** Association of pNfL concentrations with PSQI.

	Univariable analysis		Multivariable analysis	
Variable types (*n* = 46)	β (95% CI)	*p*-value	β (95% CI)	*p*-value
pNfL	1.02 (1.01–1.04)	0.003	1.04 (1.01–1.07)	0.007
Hemorrhage location	1.14 (0.44–2.97)	0.80	3.09 (0.36–26.81)	0.31
Ventricular extension	11.25 (1.90–66.73)	0.08	41.91 (2.01–874.29)	0.02
Surgical hematoma	0.24 (0.04–1.66)	0.15	0.09 (0.001–5.26)	0.24

## Discussion

Patients with intracerebral hemorrhage (ICH) frequently experience unpredictable long-term deficits, including sleep disorders (19). The absence of specific and sensitive biomarkers for predicting sleep disorders post-ICH remains a critical issue. Neurofilament light chain (NfL), a protein extensively expressed in neuronal axons (20), has been identified as a biomarker of some neurological diseases (15).

In this study, we reported Glasgow Coma Scale (GCS) scores upon admission, age, ICH volume, ICH location, and plasma NfL levels measured at corresponding time points post-ICH in a cohort of 49 patients. We observed that the elevated levels of pNfL in ICH patients, compared to healthy controls, underscore its potential as a biomarker for neuronal injury. This observation suggests that NfL is released into the bloodstream following neuronal damage. Our findings are consistent with previous research, indicating that NfL serves as a marker for the extent of brain injury (21). The longitudinal elevation in pNfL levels within the first week following ICH suggests the presence of secondary neurological impairment, with pNfL production outpacing its metabolism during this critical period. Potential contributing factors of production possibly include surgical intervention and postoperative cerebral edema. With surgical intervention to remove intracranial hematoma, some normal brain tissue (including neuroaxons) on the surgical path will be damaged. At the same time, postoperative cerebral edema might cause local brain tissue displacement to lead the traction and injury of neural axons. Previous study established that NfL undergoes renal excretion, with urinary NfL concentrations exhibiting a direct proportionality to serum levels in individuals with intact renal function (22). The factor of metabolism possibly includes impaired renal function from electrolyte imbalances, and low renal perfusion, which reduce the speed of pNfL exclusion *in vitro* after ICH. The area under the curve (AUC) values from both admission and day 3 post-ICH exceeded 0.9. Although the AUC for pNfL on day 3 post-ICH was higher than at admission, the admission AUC was deemed more clinically significant due to its superior timeliness. In the acute phase, pNfL levels measured upon admission were negatively correlated with GCS scores and positively correlated with hematoma volume. These correlations suggest that pNfL levels are indicative of injury severity. In the chronic phase, the functional recovery significantly impacted the quality of life in patients with ICH. Our findings indicated that the mean pNfL levels during hospitalization were negatively correlated with the Glasgow Outcome Scale Extended (GOSE) scores at 6 months post-ICH and positively correlated with the Pittsburgh Sleep Quality Index (PSQI) scores. This suggests that pNfL levels are predictive of both prognostic recovery and sleep quality at 6 months following ICH. Furthermore, hematoma volume at admission was found to be strongly correlated with clinical outcomes in ICH patients (23, 24). In this study, we investigated the accuracy of pNfL, hematoma volume, and their combination in identifying sleep disturbances. Our findings indicated that the combined use of pNfL and hematoma volume exhibited superior predictive performance compared to hematoma volume alone.

Our study has several limitations. Firstly, the patient data were collected from a single hospital, which may limit the generalizability of the findings. Secondly, the relationship between pNfL levels and clinical outcomes may not be directly evident without measuring NfL levels at 6 months post-ICH. Additionally, due to the study’s design, clinical outcome scores were predominantly assessed via telephone interviews. Nonetheless, our results also demonstrated the potential utility of plasma NfL as a biomarker for predicting certain chronic outcomes of ICH during hospitalization. Additionally, plasma NfL served as a valuable complement to hematoma volume in assessing ICH severity and forecasting its prognosis.

## Data Availability

The original contributions presented in the study are included in the article/supplementary material, further inquiries can be directed to the corresponding author.
